# Natural Language Processing for Identification of Hospitalized People Who Use Drugs: Cohort Study

**DOI:** 10.2196/63147

**Published:** 2025-07-18

**Authors:** Taisuke Sato, Emily D Grussing, Ruchi Patel, Jessica Ridgway, Joji Suzuki, Benjamin Sweigart, Robert Miller, Alysse G Wurcel

**Affiliations:** 1 Tufts Medical Center, Tupper Building 4F, 800 Washington St, Boston, MA, United States, 1 617 636 4605; 2 University of Chicago School of Medicine, Chicago, IL, United States; 3 Brigham and Women's Hospital, Boston, MA, United States

**Keywords:** SIRI, natural language processing, NLP, people who use drugs, substance use disorder, HIV, hepatitis C, HCV, substance use, readmission, mortality, assessment, cardiovascular disease, drug use, electronic medical record, serious injection-related infections

## Abstract

**Background:**

People who use drugs (PWUD) are at heightened risk of severe injection–related infections. Current research relies on billing codes to identify PWUD—a methodology with suboptimal accuracy that may underestimate the economic, racial, and ethnic diversity of hospitalized PWUD.

**Objective:**

The goal of this study is to examine the impact of natural language processing (NLP) on enhancing identification of PWUD in electronic medical records, with a specific focus on determining improved systems of identifying populations who may previously been missed, including people who have low income or those from racially and ethnically minoritized populations.

**Methods:**

Health informatics specialists assisted in querying a cohort of likely PWUD hospital admissions at Tufts Medical Center between 2020‐2022 using the following criteria: (1) *ICD-10* codes indicative of drug use, (2) positive drug toxicology results, (3) prescriptions for medications for opioid use disorder, and (4) applying NLP-detected presence of “token” keywords in the electronic medical records likely indicative of the patient being a PWUD. Hospital admissions were split into two groups: highly documented (all four criteria present) and minimally documented (NLP-only). These groups were examined to assess the impact of race, ethnicity, and social vulnerability index. With chart review as the “gold standard,” the positive predictive value was calculated.

**Results:**

The cohort included 4548 hospitalization admissions, with broad heterogeneity in how people entered the cohort and subcohorts; a total of 288 hospital admissions entered the cohort through NLP token presence alone. NLP demonstrated a 54% positive predictive value, outperforming biomarkers, prescription for medications for opioid use disorder, and *ICD* codes in identifying hospitalizations of PWUD. Additionally, NLP significantly enhanced these methods when integrated into the identification algorithm. The study also found that people from racially and ethnically minoritized communities and those with lower social vulnerability index were significantly more likely to have lower rates of PWUD-related documentation.

**Conclusions:**

NLP proved effective in identifying hospitalizations of PWUD, surpassing traditional methods. While further refinement is needed, NLP shows promising potential in minimizing health care disparities.

## Introduction

In the absence of harm reduction tools, people who use drugs (PWUD) are at increased risk of disease, hospitalization, and death [1-3]. Gaps in the provision of guideline-concordant care to hospitalized PWUD occur, especially among individuals from racially and ethnically minoritized communities [4-6]. Barriers to optimization of health care for hospitalized PWUD include undertreatment of pain and substance use disorders, which have been linked to discharges before medical optimization and higher rates of readmission and mortality [7-9]. Best practices for managing PWUD in a hospitalized setting include addiction care itself as well as treatment and prevention of life-threatening infections [10].

Effective identification of hospitalized PWUD is essential for epidemiological tracking, resource allocation, and evaluating interventions. However, current methodologies often fail to accurately capture this population. The “gold standard” for identifying PWUD hospitalizations is human-guided chart review, a highly regulated and time-intensive process with potential consequences for breach of confidentiality [11,12]. Administrative billing codes (also known as International Classification of Disease codes, ICD codes) have been used for PWUD identification. Unlike several other common conditions such as cardiovascular diseases for which *ICD-10* codes are highly accurate [13,14], a systematic review found that for identification of PWUD, *ICD-9/10* codes had high specificity but limited sensitivity ranging from 47%‐83% [15,16]. Indicators for substance use tend to be noted in the social history section of the electronic medical record (EMR) rather than a formal diagnosis. Some researchers have used the hepatitis C virus (HCV) codes as a marker of drug use, although there are a substantial number of people with HCV who do not currently use drugs or have ever used drugs [16,17].

The barrier to identifying PWUD can potentially be addressed with natural language processing (NLP), to leverage artificial intelligence (AI) algorithms for interpretation of the written text in a context-relevant manner [18]. NLP has been effectively applied to medical examiners’ reports to increase the accuracy of identifying substance use disorder-related deaths [19], identify substance use disorders in outpatients with HIV [20], and enhance preventive care for hospitalized patients with HIV [17]. In particular, regular expression (RegEx), a rule-based text-matching framework, has been used to identify text patterns [21]. RegEx has recently been used as a tool for identification of encounters with people with opioid use disorder (OUD) [22]. A few studies have examined the application of NLP to identify hospitalized PWUD admitted for bloodstream infections; however, these efforts were single-center evaluations, focused only on injection drug use [23-26]. Despite its innovative capacity to identify PWUD, the field of NLP methodology is nascent. The goal of this study was to evaluate the impact of NLP on the creation of a cohort of hospitalized PWUD and to evaluate disparities in documentation.

## Methods

### Definition of PWUD

As “drug use” is a broad term, it is worth emphasizing that “PWUD” in this study includes the use of cocaine, methamphetamine, fentanyl, and heroin. We use the term PWUD to describe people in the cohort, rather than “people who inject drugs”—another term used to describe this population—because these drugs can be consumed intravenously, smoked, or snorted. We do not use the term substance use disorder (SUD), as some PWUD do not meet diagnostic criteria for SUD and may not identify as having an SUD. Although drug use can also include cannabis and alcohol, we did not include these substances in the definition of drug use.

### Overview of Cohort Creation

Tufts Medical Center (TuftsMC) is a tertiary health care center located in Boston, Massachusetts, with a strong history of clinician-researcher partnerships to improve care for PWUD [5,27,28]. A health informatics specialist (RM) queried hospitalizations likely involving PWUD at TuftsMC between January 1, 2020, and April 1, 2022, guided by specific criteria (see below). The unit of measurement was hospitalization encounters, not individual patients, even if from the same patients, which requires separate clinical considerations and presents a distinct opportunity for the implementation of evidence-based practices such as introducing medications for OUD.

The presence of any of the following criteria (ie, abbreviated with the letters B, D, M, and N) were used to qualify the hospitalizations for inclusion in the PWUD cohort:

- B (Biomarkers): In line with a previous study, positive urine toxicology for drugs or medications for SUD (eg, cocaine, amphetamine, methadone, suboxone, fentanyl, opiate, oxycodone), positive HCV antibody with positive or quantifiable HCV viral load [29]
- D (Diagnostic codes): Presence of *ICD-9* and or *ICD-10* code for overdose, substance use disorders, substance-related disorders, and Hepatitis C, considering historical diagnoses and those retained in EMRs and inactivated diagnoses that did not migrate with the transition
- M (Medications for opioid use disorder): Sublingual buprenorphine (suboxone or subutex) or oral methadone listed as medications in outpatient medication reconciliation, given during hospitalization, or prescribed at discharge. We noted that methadone for OUD is not a medication prescribed at discharge, but is included via discharge reconciliation [30].
- N (Natural language processing): An iterative list of keywords that are commonly used to describe PWUD in EMR (Table 1, Textbox 1) was refined by the study team and then provided to the health informatics specialists [31]. The RegEx patterns were used to identify keywords in the EMRs, accounting for misspellings and variations in context, with incorporation of tokenizing and parsing syntax, context embedding, and approximate string matching. These features enabled context-specific word detection that accounted for minor misspellings or aggregated words. The algorithm was run on the entire EMR, including but not limited to nursing notes, physician notes, discharge summaries, and emergency room records.

**Table 1.** List of *ICD-9/10* Codes for inclusion into PWUD cohort.

Parent code	Description
*F11*	Opioid-related disorders
*F14*	Cocaine-related disorder
*F15*	Other stimulant–related disorders
*T400-T406; T436*	Poisoning by opium, heroin, other opioids, methadone, synthetic narcotics, cocaine, unspecified narcotics and psychostimulants.
*0.70.41, 070.44, 070.51, 070.54, 070.70, 070.71*	Hepatitis C
*B18.2*	Chronic viral hepatitis C

Textbox 1. **List of words programmed into NLP to detect PWUD encounters.**

IVDU, FENTANYL, Methadone, heroin, suboxone, IVDA, drug abuse, SUD, Substance use disorder, opioid use disorder, opioid abuse, OUD, opioid overdose, illicit drugs, addicted, addict, drug addict, injection drug use, intravenous drug use, uses fentanyl, Uses heroin, PWID, abuses drugs, injects heroin, injects drugs, injects fentanyl.

In addition to the above data, each encounter also had linked demographics data (eg, age, race, ethnicity, gender), length of hospitalization, and social vulnerability index (SVI). The SVI is a tool developed by the Centers for Disease Control and Prevention, used to assess the community’s susceptibility to disasters and emergencies; it uses 16 census-based data points to help assess local communities’ need for aid before and after the disaster [32]. It evaluates factors such as socioeconomic status, disability, minority status, and areas that may need additional support during crises. It is a holistic way to represent the social and economic stability of neighborhoods. The SVI was provided as a quartile (eg, 1, 2, 3, 4), with 1 representing the highest level of social vulnerability. Using the Stata software (version; StataCorp), we examined the association between key indicators (ie, race and SVI) and the level of documentation for SUDs.

### Data Analysis

Hospitalizations were classified based on the combination of domains (ie, B, D, M, N). A percentage of charges from the D-only and N-only group was selected for chart review by two research members (EDG, TS). The number of charts reviewed was determined by feasibility and proportion to the entire cohort. Coders reviewed each chart for information that indicated drug use (excluding alcohol and cannabis). The process for determining whether a hospitalization event occurred with PWUD included: (1) assessing three types of notes in each chart—emergency department admission note, history of present illness, and discharge summary and (2) using the Epic search bar—a tool that allows for keyword search within a person’s EMR profile—for keywords (Textbox 1).. The coders conducted intercoder reliability testing after completing their first 20 chart reviews, which showed consistency. A logistic regression was performed to examine factors for drug use associated with high documentation, introduced into the cohort by the presence of all of the 4 domains (B, D, M, N) versus low documentation (NLP only).

### Ethical Considerations

The study has been approved by the Health Sciences Institutional Review Board of the TuftsMC with waiver of consent granted (approval no. 2450). Identifiable data was only accessed by IRB approved study staff with approrpiate training. Identifiable data was stored on a secure file. As this was a retrospective study, there was no compensation provided to the cohort.

## Results

**Figure 1.**
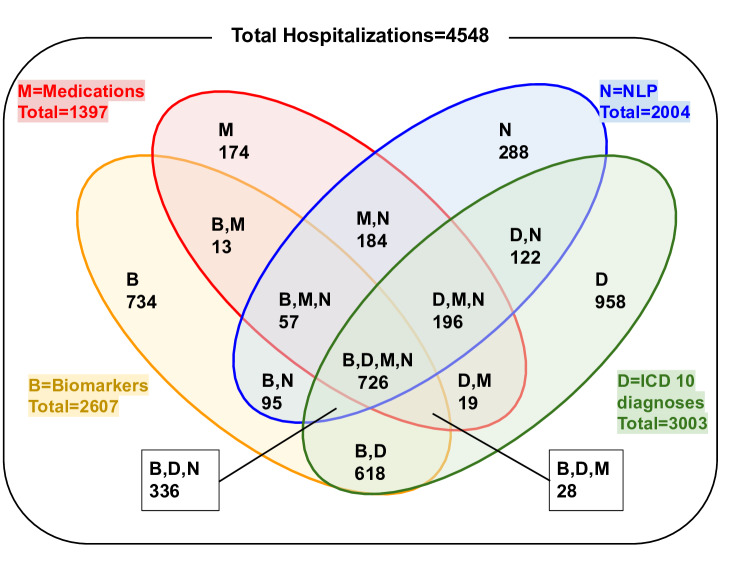
Venn diagram illustrating the total number of hospitalizations in each cohort. B: biomarkers; D: diagnostic codes; M: medications for opioid use disorder; N: natural language processing.

The Venn diagram illustrates how 4548 hospitalizations involving PWUD entered the cohort based on inclusion criteria (Figure 1). The study participants’ characteristics are shown in Table 2, along with results of the multivariable logistic regression. People who identified as White or non-Hispanic had higher odds of entering the cohort through NLP alone (adjusted odds ratio [aOR]=2.07; 95% CI 1.54, 2.79). Notably, individuals from the most socioeconomically disadvantaged quartiles (1st and 2nd SVI quartiles) were also significantly more likely to enter the cohort through NLP alone (aOR=1.41; 95% CI 1.06, 1.88). The subcohorts with the highest number of hospitalizations were those with ICD codes only (D-group, n=958), biomarkers only (B-group, n=734), and NLP with all four criteria (B, D, N, M group, n=726). Approximately 10% (n=93) individuals in the D-only group and 35% (n=99) in the N-only group underwent chart review. As shown in Table 3, the positive predictive value (PPV) of the NLP-only cohort was 54%, outperforming the diagnostic codes-only cohort, which had a PPV of 43%. This demonstrates NLP’s ability to enhance identification of PWUD hospitalizations beyond traditional methods.

**Table 2.** Descriptive analysis of PWUD cohort and factors associated with entering the cohort as highly-documented (BDMN^a^) or minimally-documented (NLP^b^ only).

Variables	Criteria for entering cohort			Unadjusted OR^c^ (95% CI)	Adjusted OR (95% CI)
	Encounters (N=4548)	Encounters-BDMN (n=726)	Encounters-NLP^b^ only (n=288)		
Age (years), mean (SD)	47.9 (13.8)	43.3 (10.7)	45.6 (14.5)	–^d^	–
Sex, n (%)					
Male	2837 (62.4)	457 (62.9)	155 (53.8)	–	–
Female	1711 (37.6)	269 (37.1)	133 (46.2)	–	–
Race/Ethnicity, n (%)					
Racially/Ethnically minoritized^e^	1583 (34.8)	176 (24.2)	114 (60.4)	1.00 (Ref)	1.00 (Ref)
Black	773 (17)	–	–	–	–
Hispanic	469 (10.3)	–	–	–	–
Asian	122 (2.7)	–	–	–	–
Asian Indian	24 (0.5)	–	–	–
Hawaiian	1 (0.02)	–	–	–	–
Other	22 (0.5)	–	–	–	–
Unknown	172 (3.8)	–	–	–	–
White/non-Hispanic	2965 (65.2)	550 (75.8)	174 (39.6)	2.04 (1.53, 2.73)	2.07 (1.54, 2.79)^e^
Length of hospitalization, mean (SD)	38.7 (26.3)	41.5 (25.7)	34.7 (26.4)	–	–
Social variability index (quartiles)					
3rd-4th	2462 (54.1)	461 (63.5)	163 (56.6)	1.34 (1.01, 1.77)	1.41 (1.06, 1.88)^f^
1st-2nd	2070 (45.51)	262 (36.1)	124 (43.1)	1.00 (Ref)	1.00 (Ref)
Missing	16 (0.4)	3 (0.4)	1 (0.4)	–	–
Urine toxicology, n (%)					
Opiate	658 (14.5)	136 (18.7)	0	–	–
Fentanyl	1313 (24.9)	430 (59.2)	0	–	–
Oxycodone	369 (8.1)	66 (9.1)	0	–	–
Methadone	272 (5.9)	224 (30.9)	0	–	–
Cocaine	622 (13.7)	258 (35.5)	0	–	–
Amphetamine	323 (7.1)	153 (21.1)	0	–	–
Primary language, n (%)					
English	4296 (94.5)	703 (96.8)	270 (93.8)	–	–
Spanish	123 (2.7)	23 (3.2)	11 (3.8)	–	–

a BDMN: All criteria for entry into the cohort satisfied.

b NLP: natural language processing.

c OR: odds ratio.

d Not applicable.

e Multivariable model adjusted for age, sex, and social variability index.

f Multivariable model adjusted for age, sex, and race.

**Table 3.** Positive predictive values of NLP-only^a^ cohort and ICD-only^b^ cohorts.

Cohort	Hospitalizations in the cohort, n	Charts reviewed, n	Charts confirmed as true PWUD^c^ by chart review, n	Positive predictive value (%)
D (diagnostic codes present)	958	93	40	43
N (NLP present)	288	99	53	54

a NLP: natural language processing.

b ICD: International Classification of Disease codes.

c PWUD: people who use drugs.

## Discussion

Our study augments previous work by integrating NLP with diverse identification methods, including urine toxicology and medication records, while simultaneously addressing observed demographic disparities in documentation [23]. NLP has the potential to uncover hospital encounters with PWUD that may have previously been missed. Although NLP had greater PPV than diagnostic codes, its PPV remained low. We found that PWUD from racially and ethnically minoritized communities and those who had low income were more likely to be represented in the minimally documented cohort (ie, entry with NLP-only), rather than the maximally documented cohort.

Largely a result of stigma and racism, PWUD still do not have universal access to evidence-based treatment. Black PWUD tend to enter treatment with a more severe prognosis compared to their White counterparts, partly due to economic barriers in accessing treatment earlier [33]. Black, Latino, and Native American individuals also face additional challenges in accessing treatment for SUD due to geographic barriers, health care access, and potential community characteristics or rapport with clinicians [34]. We found that such a lack of rapport may be represented at the level of documentation for SUD; lack of SUD documentation was strongly associated with racially or ethnically minoritized identity (aOR=2.07).

Identification of PWUD who access medical care is important for several reasons. Best practice guidelines for hospitalized PWUD include management of substance use disorder, pain, and acute infection, testing and management for HIV and HCV, vaccinations for hepatitis or other relevant infections, and prevention of HIV with medications [10,35]. In this study, we applied NLP retrospectively. Following previous studies that identified low HIV testing rates, we plan to use NLP to augment PWUD cohort creation in a study examining patterns of HIV testing [27,36]. NLP could indeed become a valuable tool for identifying PWUD before discharge, facilitating intervention during hospitalization if EMRs could use NLP to trigger clinical decision support tools that trigger clinicians to consider SUD treatment, prescribe overdose prevention medications at discharge, order labs to prepare for pre-exposure prophylaxis, or offer vaccine services.

As we consider this study in the larger context of improving health equity, we believe that the next step would be refining the NLP system by adding more keywords, including and excluding certain conditions and medications, and conducting analyses on false positives and false negative cases. This study should be replicated in other medical centers across the United States; its wider application across various hospitals, encapsulating diverse populations and regions, will be instrumental. This study also has multifaceted applications, spanning epidemiological tracking, optimizing hospital resource utilization, and influencing the design of specific interventional studies. This study’s findings could serve as a launchpad for integrated care for PWUD with less prejudice and inequity. ReGex is a relatively fundamental AI technology, and as more advanced NLP tools become available, we envision our methodology being expanded alongside these too. Regardless of type and complexity of NLP technology, the cohorting and comparative analysis outlined in this paper can be used as a framework to assess the NLP’s performance against conventional ways of locating PWUD.

This study is not without its limitations. The NLP system, despite its effectiveness, occasionally misidentifies certain keywords. The constant calibration of the algorithm and frequent addition of keywords is needed to optimize and sustain accuracy. There are potential flaws in our characterization of domains; limitations include false positives from using ’amphetamine’ as a keyword, which unintentionally classified patients prescribed amphetamines for attention-deficit/hyperactivity disorder as PWUD. Similarly, methadone prescribed for pain management in conditions such as sickle cell disease was misclassified as OUD treatment. Achieving a balance between NLP’s inclusivity and exclusivity presents a significant challenge for this purpose. Future steps should include evaluating the NLP system’s sensitivity and specificity and iterating on the model to enhance these metrics. This will involve refining the keyword list for PWUD, enhancing the NLP algorithm to better account for common confounding variables. The field of addiction medicine is innovative and adaptive; to make NLP a meaningful clinical or research tool in this field, the NLP systems need to receive extensive training and constant input of nuanced decision-making that clinicians partake in daily. Thus, a feedback mechanism and fine-tuning to train the NLP model based on clinician feedback would be critical, fully leveraging repetitive learning, which is one of AI’s biggest strengths. Furthermore, the single-cohort design of the study may limit generalizability; therefore, future studies with streamlined cross-institutional protocols, allowing simultaneous data collection from diverse locations, would improve external validity. This study had a particular focus on comparing diagnostic codes and NLP as single identifiers of PWUD. While NLP identified PWUD with higher PPV than the diagnostic codes, it must be noted that diagnostic criteria still exceeded NLP in the actual number of PWUD cases identified. One major purpose of NLP in PWUD identification is to identify cases that are otherwise missed in conventional screenings; thus, the fact that NLP alone identified a comparable number of PWUD to diagnostic code, with a higher predictive rate, is still remarkable. Future investigation should include a more robust performance comparison between a combination of two or more PWUD clinical identification tools.

The ethics of improving identification of PWUD requires careful consideration. Medical records indicating drug use may become a source of discrimination, compromise job security, housing, and ability to care for family. To mitigate these risks, institutions should implement strict policies ensuring that NLP findings are used solely for improving patient care. Members of this research team collaborated with a broad group of experts including people with lived experience of SUD on a study outlining some of the potential pros and cons of improving systems to identify PWUD with the creation of an additional *ICD-10* code for injection drug use [37]. Future work should proactively incorporate the perspectives of individuals with lived experience of SUD. Furthermore, broader discussion regarding AI’s role in health care is needed for effective, ethical, and productive clinical implementation: “Should NLP be a “wide net” or “precision tool” when locating PWUD and connecting them to the care they need?”

Despite these limitations, we believe that this study helps frame the future of systems for measuring health care delivery to PWUD. Hospitalization represents a crucial opportunity when nonjudgmental, trauma-informed, culturally competent care can be offered to PWUD. This presents many potential applications for NLP to be built into systems that track epidemiology and inform quality improvement and implementation science. By integrating NLP, we can advance equitable PWUD care.

## Abbreviations

AI: artificial intelligence
aOR: adjusted odds ratio
EMR: electronic medical record
HCV: hepatitis C virus
ICD: International Classification of Disease codes
NLP: natural language processing
OUD: opioid use disorder
PPV: positive predictive value
PWUD: people who use drugs
RegEx: regular expression
SUD: substance use disorder
SVI: social variability index
TuftsMC: Tufts Medical Center
